# Mol2Image: an enhanced DDI prediction framework leveraging drug molecular descriptors

**DOI:** 10.1186/s12859-026-06552-7

**Published:** 2026-07-22

**Authors:** Nourhan Helmy, Huda Amin Maghawry, Nagwa Badr

**Affiliations:** Information Systems Department, Faculty of Computer and Information Sciences, Ain Shams University, Cairo, Egypt

**Keywords:** Drug–drug interactions (DDIs), Deep learning in drug discovery, SMILES, Chemical structures, Molecular descriptors

## Abstract

Drug–drug interactions (DDIs) are a critical safety issue in clinical practice, as they can lead to severe and often unpredictable adverse effects. This risk becomes significantly higher in multi-drug therapies, which are increasingly used in the treatment of complex and chronic diseases such as cancer, cardiovascular disorders, and diabetes. However, identifying DDIs through in vivo studies is costly and time-consuming. In this study, a novel DDI prediction model, Mol2Image, has been proposed that utilizes chemical structure features derived from Simplified Molecular Input Line Entry System (SMILES) representations, including molecular property descriptors and structural fingerprints. The proposed model combines chemical structure information with automated feature learning. Molecular descriptors and structural fingerprints extracted from SMILES representations are converted into visual patterns that capture key chemical characteristics of each drug. These images are then processed by a Convolutional Neural Network (CNN) to learn high-level structural features associated with drug–drug interactions. The model is trained and evaluated using two benchmark DDI datasets: the Drugbank dataset, which consists of 443,046 interactions, and ChCh-Miner, which consists of 48,514 DDIs. Experimental results demonstrate that the proposed model (Mol2Image) achieves competitive performance compared with several state-of-the-art methods. Experimental results demonstrate that the proposed model consistently outperforms existing approaches, achieving accuracies of 0.9608 and 0.9683 using the Drugbank dataset and ChCh-Miner dataset, respectively. Ultimately, Mol2Image provides a highly scalable, strictly structure-centric framework that ensures superior predictive accuracy with minimal computational overhead, operating entirely independently of clinical data.

## Introduction

Recently, multi-drug therapy has become a common approach for treating complex and chronic diseases such as cancer, diabetes, and heart conditions [1–4]. Moreover, most human diseases arise from complex biological mechanisms that cannot be effectively treated with a single drug [5]. Typically, polypharmacy involves two to nine drugs administered simultaneously [6–8]. However, this practice increases the risk of adverse drug reactions (ADRs), which can sometimes result in severe or fatal outcomes [9–11]. In the United States, ADRs are estimated to cost over $10 billion annually, with drug–drug interactions (DDIs) accounting for more than 30% of these expenses [12]. DDIs occur when one drug affects another, such as by changing its metabolism, absorption, or clearance. these affects are among the leading causes of emergency department visits due to unexpected adverse reactions or treatment failures [13], and are typically classified into three categories: synergistic, where the combined effect of two drugs is greater than the sum of their individual effects; antagonistic, where the interaction reduces or counteracts the effectiveness of one or both drugs; and neutral, where co-administration does not produce any meaningful change in therapeutic effect [14]. Many of these interactions are rare and not detected in clinical trials, making manual identification both difficult and inefficient [15]. Moreover, ADEs caused by DDIs are among the leading reasons for drug withdrawals from the market [16]. Therefore, accurate detection of DDIs is critical. However, identifying them through in vitro or in vivo experiments is often time-consuming, complex, and costly [17], highlighting the need for efficient computational alternatives that can support timely and safe treatment decisions. Recently, researchers have gathered drug data from literature, reports, and other sources to build public databases [18–21], which have supported the advancement of computational prediction methods. Also, there are several machine learning and deep learning-based approaches that have been proposed for predicting DDIs [22, 23]. Computational methods for DDI prediction are typically categorized into four main groups: text-based [24], network-based [25], similarity-based [17], and structure-based [26] approaches. Text-based methods mine biomedical literature to extract relationships between drug pairs and predict potential DDIs based on drug descriptions [27]. Network-based methods infer interactions based on drug similarity propagation or the structure of interaction networks [28]. Similarity-based methods assume that drugs with similar properties are likely to interact with the same drug and that similar molecular structures typically confer similar biological properties [26, 29, 30]. These methods usually calculate similarity using metrics such as the Jaccard coefficient [31]. Structure-based methods predict DDIs using the molecular structure of drugs without relying on external drug descriptions, thereby improving both efficiency and accuracy. Structure-based methods rely solely on the molecular structure of drugs, typically encoded as SMILES (Simplified Molecular Input Line Entry System) [26], which is a standardized set of grammar rules and symbols used to represent the atoms and structure of a chemical compound. However, sequence-based features have limitations, as they fail to fully capture the two- or three-dimensional spatial structures of molecules and often overlook important topological characteristics. SMILES translate chemical information into numerical form, making it suitable for mathematical and computational modeling. Deep learning architectures such as Convolutional Neural Networks (CNNs) [32], Recurrent Neural Networks (RNNs) [33], and Graph Neural Networks (GNNs) [34] are capable of automatically learning hierarchical and abstract representations from raw input data, including Simplified Molecular Input Line Entry System (SMILES) sequences, molecular structures, and drug similarity matrices. While most existing models primarily depend on available data for approved drugs, Mol2Image focuses on leveraging the chemical structure of the drug and uses molecular descriptors. Molecular descriptors [35] play a crucial role in disciplines such as chemistry, pharmaceutical sciences, environmental policy, health research, and quality control. ‹›They enable the transformation of molecules from physical entities into numerical representations, allowing for mathematical analysis of their underlying chemical information. These descriptors can be broadly divided into two categories: experimental measurements, which include values such as the logarithm of the octanol/water partition coefficient (log P), molar refractivity, dipole moment, polarizability, and other additive physicochemical properties, and theoretical descriptors, which are derived from symbolic representations of molecules and can be further classified based on the specific type of molecular representation employed. Theoretical descriptors are classified into four main types. Constitutional and count descriptors are the simplest, derived directly from the molecular formula, and include features such as the number and types of atoms and the molecular weight. Physicochemical property descriptors capture characteristics like molecular size, shape, and electronic distribution; however, their accuracy often depends on database completeness, and missing data fragments can reduce reliability. Structural descriptors describe three-dimensional conformational properties, such as intramolecular hydrogen bonding, typically obtained through computational analysis. Finally, connectivity (Chi) indices are numerical values that encode molecular topology, representing how atoms are connected and aiding in the prediction of molecular properties. Importantly, each molecular descriptor reflects only a specific aspect of the overall chemical information contained in a real molecule. In addition, the proposed model, Mol2Image, employs Morgan fingerprints, a type of descriptor that generates numerical values from a molecule’s representation (e.g., SMILES) to encode its structural, physicochemical, and topological properties. Mol2Image uses a CNN as its core component. CNNs can automatically extract features through representation learning, enabling the model to capture hierarchical patterns directly from raw input data without relying on manual feature engineering [36]. Unlike previous methods, Mol2Image systematically evaluates and integrates an optimal 21-descriptor set alongside Morgan fingerprints, maximizing the extraction of essential chemical features while mitigating noise and redundancy in the generated 2D visual space. Furthermore, rather than employing deep, resource-heavy networks, Mol2Image utilizes a streamlined, Siamese-like dual-branch CNN architecture comprising only three convolutional blocks per drug input to process these images. This lightweight design significantly reduces computational overhead without sacrificing predictive performance. Extensive evaluations demonstrate that Mol2Image effectively balances this optimized feature-to-image mapping strategy with a highly efficient CNN, outperforming existing methods in terms of accuracy, F1-score, and precision. Ultimately, Mol2Image provides a scalable, strictly structure-centric solution that operates independently of clinical data availability, making it particularly advantageous for preliminary interaction screening based solely on molecular descriptors. The rest of the paper is organized as follows: Sect.  2 surveys the most relevant work related to structure-based methods for DDI prediction. Section  3 introduces the dataset used to train the proposed model, the architecture of the proposed model, and the training phases. Section  4 describes in detail the features selected to train the model and provides the experimental results of the proposed approach. Finally, Sect.  5 states the conclusion and future work.

## Related work

The proposed model, Mol2Image, adopts a structure-based approach by leveraging SMILES representations as input features. Several recent models have explored this direction. For example, the PTB-DDI framework [37] proposes a deep learning approach to predict drug–drug interactions (DDIs) using 1D SMILES strings. The framework, PTB-DDI, integrates four key components: a Chemberta [38] tokenizer for molecular representation, BiLSTM [39] to capture contextual features, a machine learning model for learning nonlinear relationships, and a final interaction predictor. It introduces two operating modes, parameter-sharing and parameter-independence, to explore different modeling strategies. The model is evaluated on two benchmark datasets, BIOSNAP [40], achieving AUC-ROC, PR-AUC, and F1 scores of 0.997, 0.995, and 0.984, compared to DrugBank’s [41] 0.896, 0.873, and 0.826. However, it suffers from relatively low prediction accuracy. StructNet-DDI [42], on the other hand, predicts drug–drug interactions by leveraging structural information from drug molecules, extracted from their SMILES representations. It combines two types of features: Morgan fingerprints [43], which encode atom connectivity and local structural environments as binary vectors, and twelve carefully selected molecular descriptors that capture physicochemical and pharmacokinetic properties such as molecular weight, logP, hydrogen bonding potential, and topological features. These features are then transformed into image-like representations, enabling the use of a convolutional neural network. The model adopts a modified ResNet18 [42] architecture, which uses residual connections to efficiently learn deep and complex feature representations while avoiding issues like gradient vanishing. By concatenating the image-based representations of two drugs and feeding them into ResNet18, the model learns to classify whether the drug pair is likely to interact. This model achieved an AUC of 99.7%, an accuracy of 94.4%, and an AUPR of 99.9%. Although this method is effective, it is computationally intensive and resource-demanding. MI-DDI [44] is a multi-view feature-based.

interpretable deep learning framework for predicting drug-drug interactions (DDIs). The method integrates atomic-view and substructure-view features to enhance prediction accuracy and interpretability. Atomic-view features are extracted using a Message Passing Neural Network (MPNN) from molecular graphs generated by RDKit [45], capturing detailed atom-level interactions. Substructure-view features are derived from SMILES strings using transformer encoders, which identify and encode recurring chemical substructures. These multi-view features are combined into a unified drug embedding matrix. An interpretable interaction module then constructs an interaction matrix to highlight relevant atom/substructure pairs, which is used to generate weight matrices for the final prediction. The framework employs a multi-layer perceptron (MLP) [46] to predict DDIs. While it enhances interpretability, it introduces significant computational overhead due to its dual feature extraction pipelines. Additionally, CASTER [47], a deep learning framework designed to predict drug-drug interactions (DDIs) by focusing on chemical substructures rather than whole-molecule representations. This method employs sequential pattern mining (SPM) to identify frequent substructures from SMILES strings, ensuring relevance to interaction mechanisms. To enhance generalizability, CASTER leverages unlabeled data (e.g., drug-food pairs) through an auto-encoder, pretraining latent embeddings to improve performance with limited labeled data. A dictionary learning module then projects these embeddings onto a substructure-based subspace, generating interpretable coefficients that highlight key substructures driving interactions. Evaluated on DrugBank [41] and BIOSNAP [40] datasets, this model achieved accuracy 0.9553, F1score 0.9563, Recall 0.9643, and a precision of 0.9645. There is DeepDDI [16], which is a deep learning-based computational framework designed to predict drug–drug interactions (DDIs) and drug–food constituent interactions (DFIs) using only drug names and their structural representations (SMILES format). DeepDDI generates human-readable output sentences describing 86 predefined DDI types with high accuracy (mean accuracy of 92.4%) by using a deep neural network (DNN) trained on a gold-standard DrugBank dataset of 192,284 DDIs. The model employs structural similarity profiles (SSPs) as feature vectors to capture molecular properties. It was successfully applied to various tasks, including identifying potential mechanisms of adverse drug events (ADEs), suggesting safer drug alternatives, predicting interactions with food constituents, and inferring bioactivities of food compounds. While existing structure-based models, such as StructNet-DDI, have demonstrated the utility of combining molecular descriptors with CNNs, they often rely on computationally intensive architectures like ResNet18 and utilize standard, limited descriptor sets. To address these limitations and establish a clear innovation boundary, the proposed Mol2Image framework introduces a highly optimized and computationally efficient approach. Another recent approach, SA-DDI [48], introduces a substructure-aware graph neural network designed to identify the key functional groups responsible for DDIs. Built upon a message-passing architecture, this model incorporates a novel substructure attention mechanism to capture size- and shape-adaptive molecular substructures. To further refine its predictions, SA-DDI employs a substructure-substructure interaction module (SSIM) that explicitly models chemical interactions by highlighting critical substructures while de-emphasizing minor ones. This methodology not only achieves high predictive performance on real-world datasets but also significantly improves model interpretability by visually detecting the specific structural components driving the interactions. Despite these architectural improvements, a prevailing challenge remains the ‘cold-start’ problem, where accurate predictions are required for novel compounds with severely limited data. To address this, recent literature has shifted towards few-shot and meta-learning paradigms. Notably, Meta3D-DDI [49] was developed to predict interactions for new drugs by leveraging 3D molecular conformations and few-shot learning (FSL). The architecture employs a 3D graph neural network with a continuous filter interaction module to capture atomic pairwise distances, ensuring rotation and translation invariance. By utilizing a bilevel optimization strategy and a rigorous scaffold-based splitting approach, the framework successfully transfers meta-knowledge to predict interactions with minimal structural data leakage. Comprehensive cross-domain benchmarks like Meta-MolNet [50] have been introduced to enhance model generalization in low-data drug discovery environments. While these state-of-the-art methods successfully tackle data sparsity through complex meta-learning and 3D graph modeling, our proposed Mol2Image framework offers a streamlined, complementary approach. By deterministically mapping an optimized set of molecular descriptors into 2D spatial images processed by a lightweight CNN, Mol2Image achieves highly accurate, clinical-data-independent predictions while significantly reducing the computational complexity typically associated with 3D or meta-learning architectures.

The proposed model Mol2Image offers a streamlined, complementary approach. By deterministically mapping an optimized set of molecular descriptors into 2D spatial images processed by a lightweight CNN, Mol2Image achieves highly accurate, clinical-data-independent predictions while significantly reducing the computational complexity typically associated with 3D or meta-learning architectures.

## Datasets and methods

Two publicly available DDI datasets have been employed to train the proposed approach. Both datasets have been widely used in prior research for benchmarking. The first one is the DrugBank dataset [37]. It contains 443,046 DDI pairs, balanced between 221,253 positive and 221,253 negative interactions. It is notably larger than many other datasets while maintaining class balance. The second one is the ChCh-Miner dataset [42]. It consists of 1514 drugs and 48,514 DDIs that have also been used in previous DDI studies. Detailed statistics on the number of pairs and drug entities for this dataset are reported in the experimental section.

### Proposed approach

**Fig. 1:**
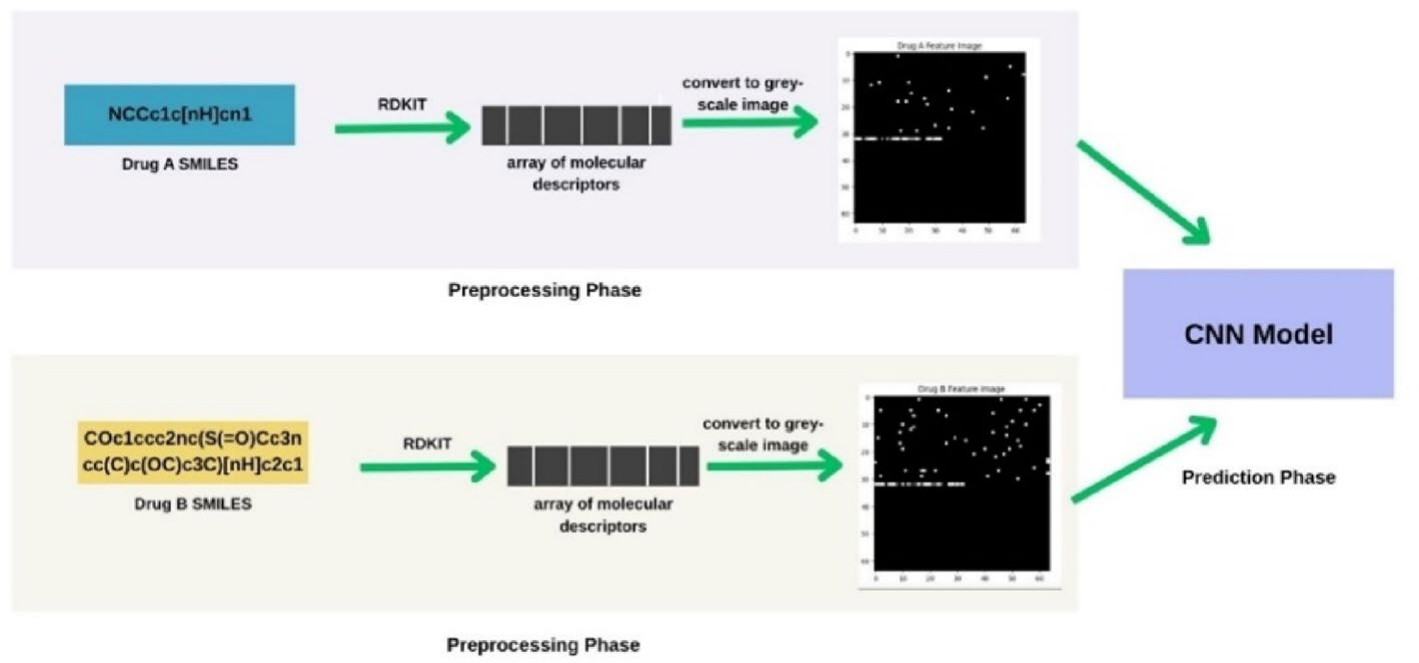
Mol2Image model architecture

The proposed model Mol2Image consists of two main phases, as shown in Fig. 1. First Phase is data preprocessing, in which Morgan fingerprints and molecular descriptors have been extracted directly from SMILES strings using RDKit, combining these features into a vector representation for model input. The second phase is the training phase using CNN as the deep learning model.

Since Convolutional Neural Networks (CNNs) can learn and exploit feature relationships more effectively than traditional models trained on tabular data [51]. Drug features have been transformed into grayscale images. This conversion enables CNNs to automatically learn complex features and recognize intricate patterns that may be difficult to capture with traditional methods [52]. By leveraging visual abstraction, CNNs can more effectively learn topological characteristics embedded in the data. Additionally, SMILES string representations have been incorporated, as models that analyze molecular chemical features tend to offer more transparent and interpretable predictions for drug-drug interactions [53].

RDKit allows us to extract a variety of molecular features, including physicochemical properties, topological descriptors, and 3D descriptors, for the proposed model Mol2Image. CNN has been used as a proposed deep learning model, and that is because CNNs outperform DNNs in feature extraction and are more effective at reducing overfitting [32].

Molecular descriptors serve as powerful quantitative tools for characterizing molecular properties, offering critical insights into structural attributes, chemical reactivity, and biological interactions. These descriptors can be efficiently computed from molecular structures using standard computational chemistry tools or obtained through higher-level quantum chemical calculations [42].

#### Data preprocessing

The proposed method does not solely rely on raw SMILES strings as its training data; rather, it utilizes them to generate a robust set of 1D chemical descriptors and Morgan fingerprints via the RDKit library. To ensure that the model captures a comprehensive representation of the molecular structure, our feature selection strategy was designed to cover diverse physicochemical categories. Initially, an extensive pool of 32 descriptors was extracted. These features were organized into four main groups alongside structural fingerprints: (1) Basic Molecular Properties, including Molecular Weight (MW), the logarithm of the octanol/water partition coefficient (LogP), Topological Polar Surface Area (TPSA), and sp3 hybridization (Prop sp^3); (2) Atomic Composition & Counts, covering the number of bonds, Hydrogen/Oxygen/Nitrogen (H/O/N) atoms, and Hydrogen Bond Donors/Acceptors; (3) Topological & Connectivity Indices, including rotatable bonds, aromatic/aliphatic/saturated rings, and Kier-Hall indices (chi0–chi4n); and (4) Structural Fingerprints, represented by the Morgan fingerprint.

While this comprehensive 32-descriptor set spans all structural aspects, empirical evaluations and visual interpretability analyses (using Gradient-weighted Class Activation Mapping (Grad-CAM)) revealed that including all features introduced computational noise and redundancy. Therefore, a rigorous ablation study was conducted to identify the optimal feature subset. The experiments concluded that a carefully selected subset of 21 descriptors effectively encapsulates the essential chemical information across all the categories without redundancy. Ultimately, these 21 descriptors, combined with the Morgan fingerprints, serve as the optimal basis for the visual pattern generation in the Mol2Image framework, enabling robust and noise-free computational modeling for DDI prediction. It is worth noting that within this optimized subset, certain descriptors, such as the number of free radicals and unpaired electrons, exhibit near-zero variance, as most approved drugs are closed-shell molecules. These features were retained to maintain a standardized extraction pipeline; their potential noise contribution is intrinsically neutralized by the CNN, which assigns negligible weights to these invariant spatial pixels during training.

#### Feature-to-image mapping strategy

**Fig. 2:**
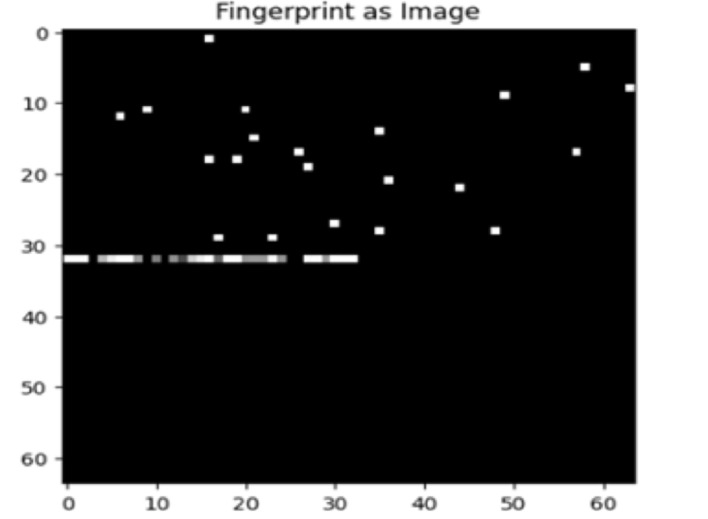
Fingerprint as an image after feature mapping

To transform the extracted 1D features into 2D structural images suitable for CNN processing, a deterministic mapping strategy was employed. First, to determine the spatial position, the data were mapped into a fixed $\:64\times\:64$ matrix (4,096 pixels). The 2048 bits of the Morgan fingerprint, representing local molecular substructures, are placed sequentially at the very beginning of the matrix, forming a contiguous structural block. Immediately following the fingerprint, the physicochemical descriptors are mapped and grouped sequentially by their chemical categories. Since the combined feature set requires only 2069 pixels, it is accommodated entirely within the 4096-pixel matrix on a strict 1-to-1 basis; thus, absolutely no dimensionality reduction is performed, and zero chemical information is lost. The remaining pixels are zero-padded, acting as a neutral background. This deliberate grouping ensures that the spatial proximity between adjacent pixels carries a genuine chemical meaning, allowing the CNN’s convolutional kernels to extract localized, chemically related patterns. Second, to determine pixel intensity, the feature values are projected into a standard grayscale color space. For the binary fingerprint bits, this translates directly to distinct active and inactive pixels. For the continuous descriptors, their values are encoded into grayscale intensities reflecting their numerical magnitude. This chemically aware spatial arrangement guarantees that the spatial hierarchies learned by the model reflect true pharmacological relationships rather than artificial data structures, as shown in Fig. 2.

#### Prediction using CNN

After the data preprocessing step is accomplished, CNNs have been used as the proposed prediction model, where input two images representing two different drugs. The model predicts whether the two drugs interact with each other. As shown in Fig. 3,

each drug image is passed through an identical CNN branch consisting of three convolutional blocks. Each block comprises a 2D convolutional layer followed by batch normalization, a ReLU activation function, and a max-pooling layer. The number of feature channels is progressively increased from 32 to 64 and then to 128, allowing the network to capture increasingly complex spatial patterns. To evaluate the proposed model Mol2Image, three feature subsets have been selected to train the proposed model Mol2Image, as shown in Table 1.

**Table 1:** Feature subsets

#	Experiment	Feature subset
1	13-descriptor experiment	No. of bonds, No. of hydrogen atoms, No. of oxygen atoms, LogP, No. of rotatable bonds, Number of hydrogen bond donors, Prop sp3, TPSA, No. of free radical, No. of Nitrogen atoms, No. of hydrogen acceptors, No. of aromatic rings, Morgan fingerprint
2	21-descriptor experiment	All features in (1) plus: Molecular weight, chi0n, chi1n, chi2n, chi3n, chi4n, chi0, chi1, No. of aliphatic rings, No. of saturated rings.
3	32-descriptor experiment	All features in (2) plus: Average ring size in the molecule, Largest ring size, Fraction of *sp*^3^ carbons, Proportion of *sp*^2^- hybridized carbons, Number of non-hydrogen atoms, Number of hetero atoms, Number of hydrogen bond acceptors, Number of atoms with unpaired electrons, Quantitative Estimate of Drug-likeness, Kier shape index, Number of atoms with unpaired electrons, Total number of rings.

**Fig. 3:**
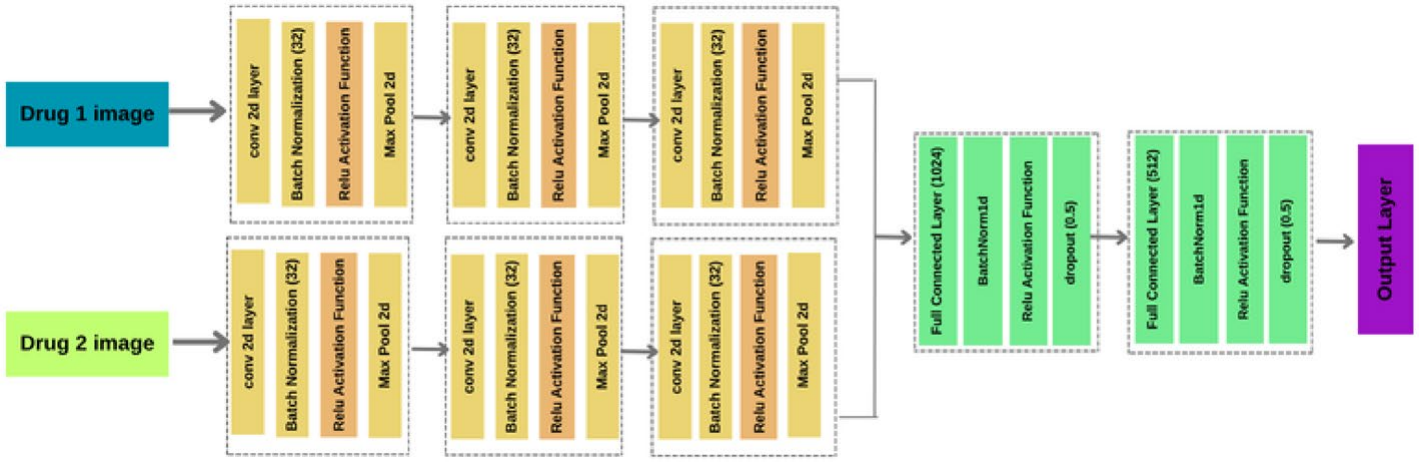
The CNN model architecture

## Experiments and results

The experiments aim to validate the proposed model, Mol2Image, and analyze its performance under different settings. The experiments are conducted in multiple stages. As mentioned in Table 2. The experimental environment had a GPU as the processor type, it had 22.5 GB of RAM, and the system RAM is 53GB. Due to the significant difference in the total number of samples between the two datasets, dataset-specific splitting strategies were employed to ensure optimal model training. For the larger DrugBank dataset, a standard 80:20 (training: testing) split was utilized. Conversely, for the smaller ChCh-Miner dataset, an approximate 90:10 split was applied to maximize the data available for the training phase, resulting in exactly 5113 unseen samples allocated for the final test set, 20 epochs, and the Adam optimizer was employed with an initial learning rate of 0.001. The performance of the proposed model has been evaluated.

**Table 2:** Model parameters

Parameters	Values
Epoch no.	20
Batch size	512
Image size	(64,64)
Optimizer	Adam optimizer
Learning rate	0.001
Processor type	GPU
GPU RAM	22.5 GB
System RAM	53.0 GB

Mol2Image uses several key evaluation metrics, including AUC-ROC, Accuracy, Precision, and F1 Score. These metrics provide a comprehensive assessment of the model’s performance. To evaluate the impact of different feature representations, multiple experiments have been conducted using varying numbers of descriptors (13, 21, and 32). The performance of each feature set was assessed using ACC, AUC-ROC, Precision, and F1-score on both the DrugBank and ChCh-Miner datasets as shown in Tables 3 and 4, respectively.

**Table 3:** Performance comparison of different descriptor feature subsets using the Drugbank dataset

Experiment	ACC	AUC-ROC	Precision	F1 score
13-descriptor experiment	0.9599	0.9908	0.9604	0.9599
21-descriptor experiment	**0.9608**	**0.9907**	**0.9612**	**0.9608**
32-descriptor experiment	0.9588	0.9906	0.9596	0.9588

Bold values indicate the best predictive performance (the highest results) achieved among all the compared models for each respective evaluation metric

**Table 4:** Performance comparison of different feature subsets using the ChCh-Miner dataset

Experiment	ACC	AUC-ROC	Precision	F1 score
13-descriptor experiment	0.9681	0.9985	0.9746	0.9697
21-descriptor experiment	**0.9683**	**0.9989**	**0.9747**	**0.9699**
32-descriptor experiment	0.9679	0.9989	0.9744	0.9695

﻿Bold values indicate the best predictive performance (the highest results) achieved among all the compared models for each respective evaluation metric

**Fig. 4:**
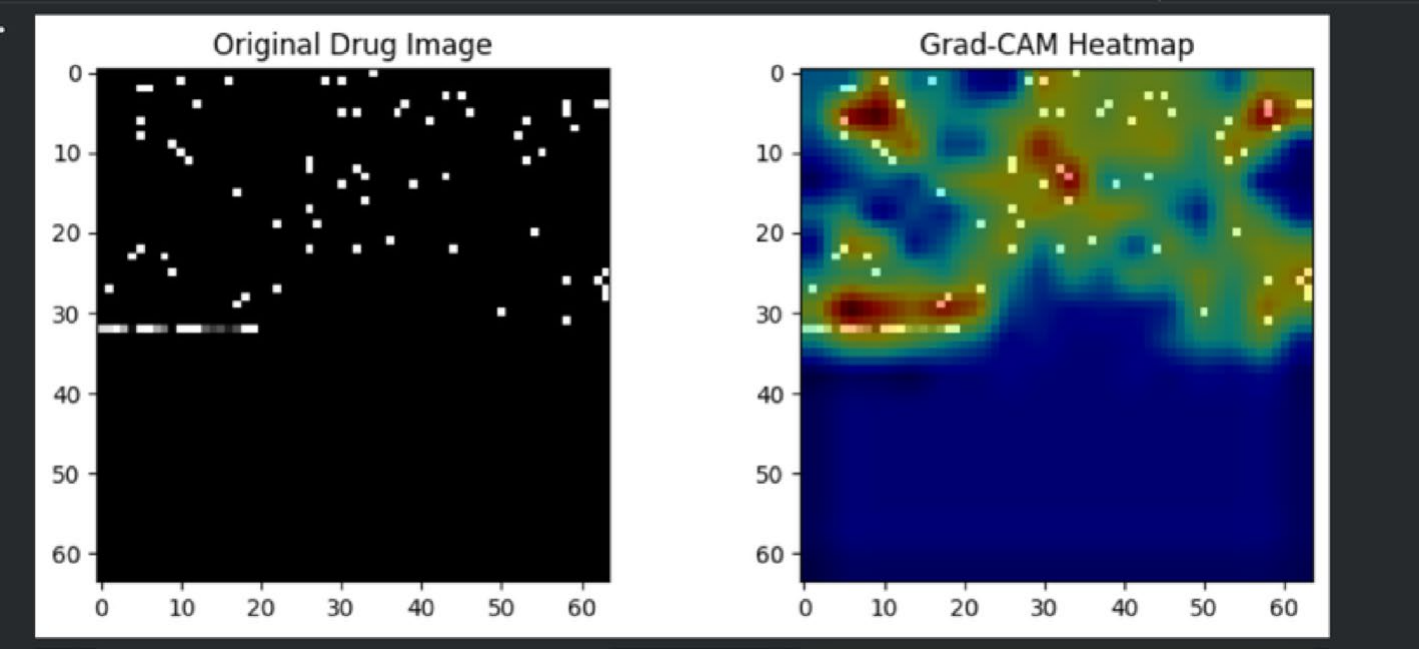
Grad-CAM visualizations illustrating the interpretability and feature focus of the Mol2Image architecture 21-descriptor model

**Fig. 5:**
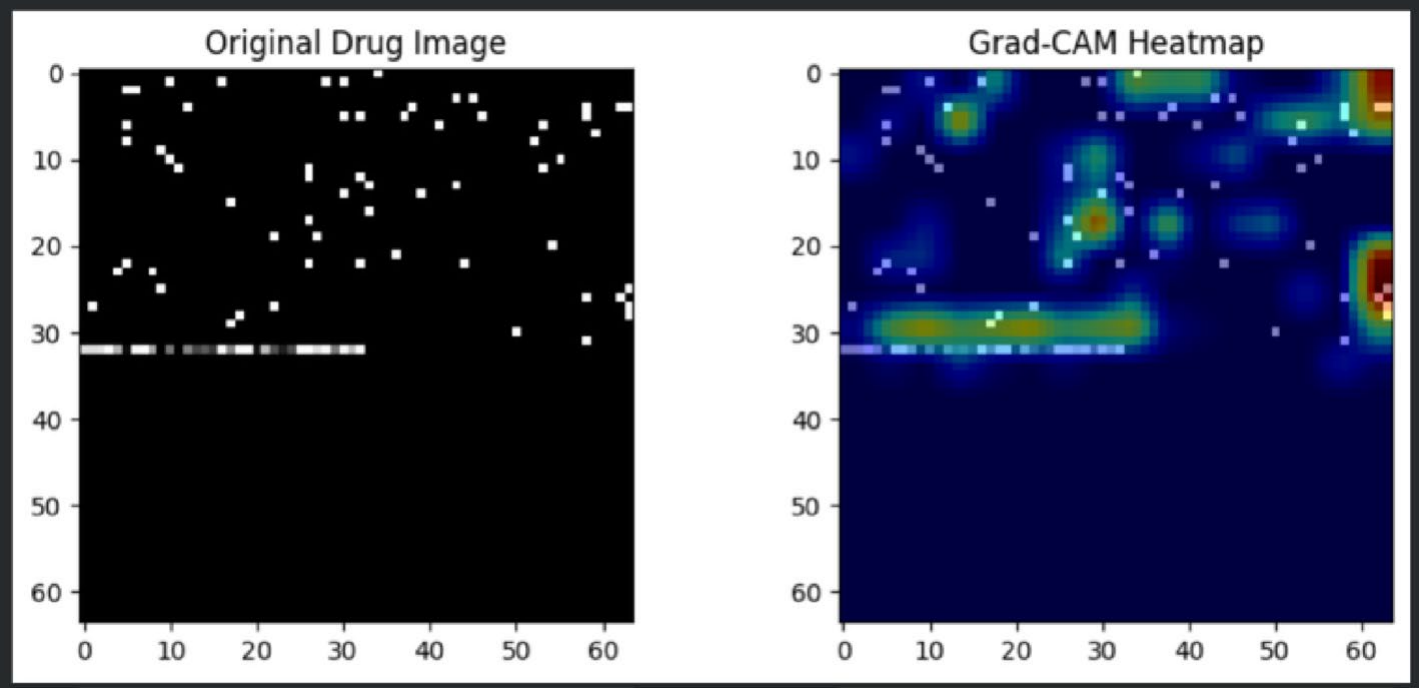
Grad-CAM visualizations illustrating the interpretability and feature focus of the Mol2Image architecture 32-descriptor model

Although the 32-descriptor feature set provides a more comprehensive representation, the experimental results show a slight degradation in performance compared to the 21-descriptor configuration. This quantitative observation is further corroborated by our visual interpretability analysis using Grad-CAM. As illustrated in Fig. 4, the optimal 21-descriptor model exhibits sharp, highly focused activation regions on critical physicochemical properties and distinct substructures within the Morgan fingerprint. In contrast, Fig. 5 reveals that incorporating the supplementary descriptors results in a more diffused and scattered activation map. This visual evidence confirms our hypothesis that adding these extra features introduces spatial noise and computational redundancy, which distract the model’s spatial attention and negatively affect generalization. Therefore, the 21-descriptor feature set was selected as the optimal configuration. Consequently, the proposed Mol2Image model is evaluated and compared to other existing approaches using this 21-descriptor subset. Also, to ensure that the performance variations between the 21-descriptor and 32-descriptor subsets were not due to random variation, a statistical significance analysis was conducted. Specifically, McNemar’s test [52] was applied to compare the paired predictions of both models on the test set. The analysis yielded a highly significant result ($\:p<0.001$), mathematically confirming that the removal of redundant features in the 21-descriptor set provides a genuine, statistically sound improvement in predictive performance rather than a random fluctuation.

The other approaches are PTB-DDI [37], CASTER [47], and 1D CNN [54]. On the DrugBank dataset, the proposed model Mol2Image outperformed PTB-DDI and CASTER and 1D CNN, achieving an accuracy of 0.9608, an AUC-ROC of 0.9907, a precision of 0.9612, and an F1-score of 0.9608, as shown in Table 5.

**Fig. 6:**
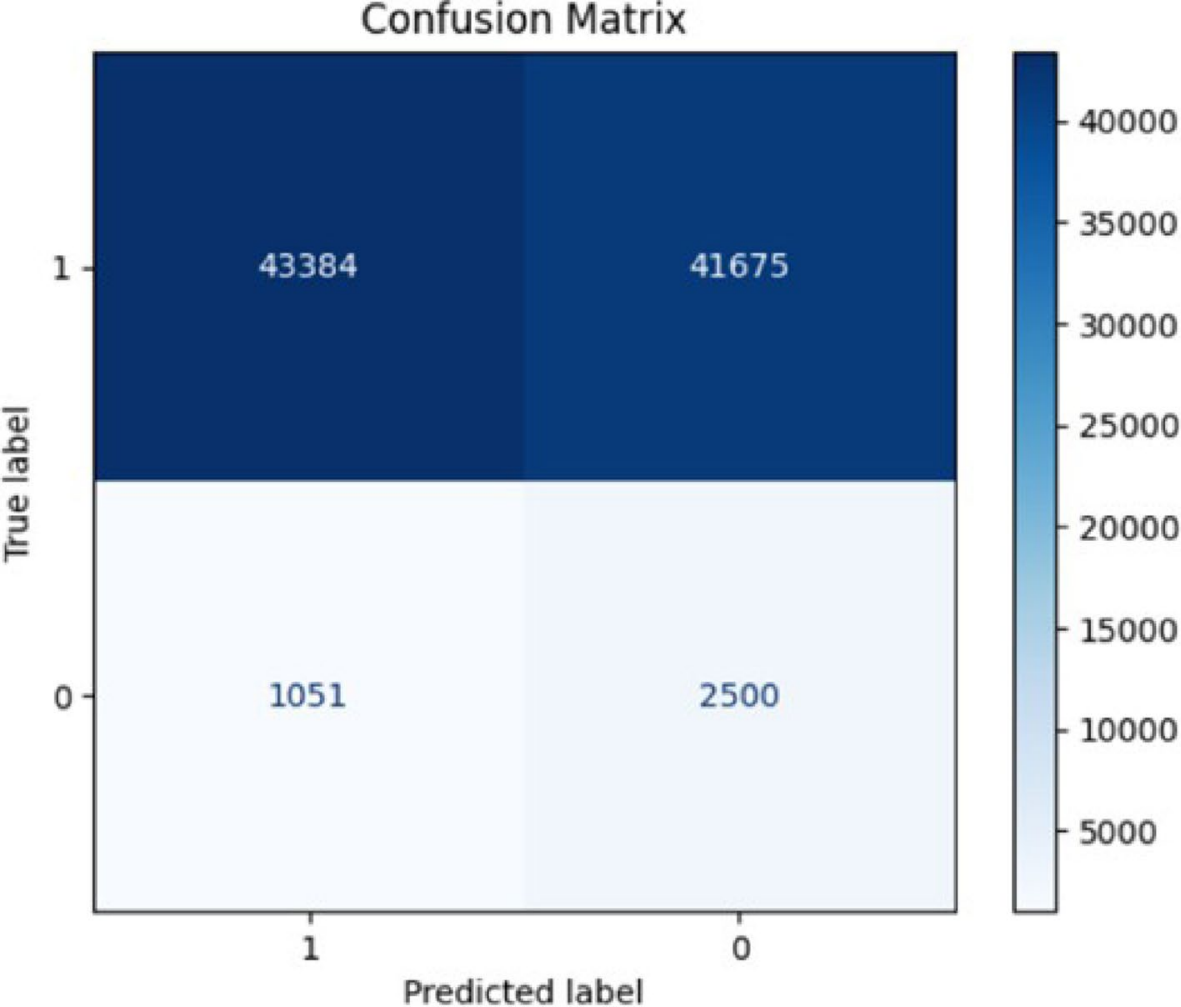
Confusion matrix of the proposed model on DrugBank dataset

**Fig. 7:**
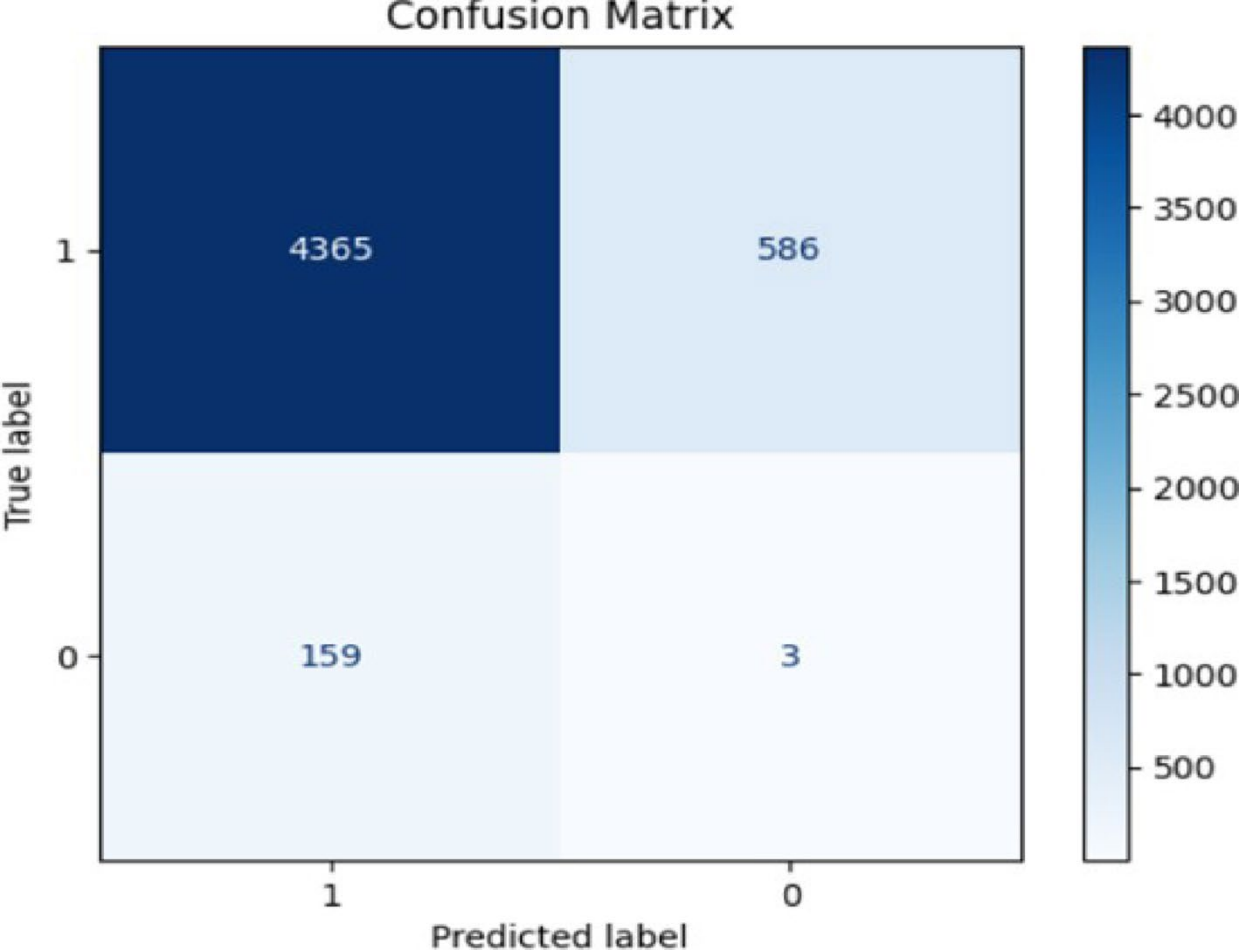
Confusion matrix of Mol2Image on ChCh-miner dataset

The confusion matrix for the DrugBank dataset is shown in Fig. 6, where it achieved 41,675 TN, 43,384 TP, 2500 FN, and 1051 FP. In contrast, CASTER, PTB-DDI, and 1D CNN achieved lower accuracies of 0.8676 and0.86 and 0.9353, respectively, with F1-scores of only 0.8767, 0.825, and 0.9369. and with precision 0.829, 0.8221, and 0.9353, also with AUC 0.9268, 0.896, and 0.9802. On the ChCh-Miner dataset, as shown in Table 5, the proposed model outperformed other approaches as it achieved an accuracy of 0.9683, slightly higher than StructNet-DDI 0.944, CASTER 0.9073, PTB-DDI 0.935, and 1D CNN 0.9492. In terms of F1 score, the proposed model Mol2Image achieved 0.9699, which is higher than StructNet-DDI, which achieved 0.967, CASTER, which achieved 0.932, PTB-DDI, which achieved 0.932, and higher than 1D CNN 0.9495. In terms of precision, CASTER achieved the highest score of 0.9993, while the proposed model Mol2Image achieved 0.9747. CASTER relies exclusively on SMILES-based chemical substructure representation, which enables highly specific pattern recognition at the structural level. This specificity significantly reduces false positive predictions, resulting in the highest precision score (0.9993). However, substructure-based approaches may be limited in capturing broader biological or pharmacological interaction signals. In contrast, the proposed Mol2Image framework integrates multimodal representations, allowing it to model both structural and contextual interaction patterns. Although this leads to a slightly lower precision (0.9747), it enhances the model’s ability to generalize and detect a wider range of true interaction pairs, while StructNet-DDI achieved 0.999, PTB-DDI 0.945 and 1D CNN 0.9529. The confusion matrix for this ChCh-Miner dataset is shown in Fig. 7, where it has 586 TN, 4365 TP, 3 FN, and 159 FP. The results of these comparisons are summarized in Table 5.

**Table 5:** Performance Comparison of the proposed model Mol2Image with existing approaches

Model	Dataset	Acc	AUC-ROC	Precision	F1 score
Mol2Image	Drugbank	**0.9608**	**0.9907**	**0.9612**	**0.9608**
1D CNN [54]		0.9353	0.9802	0.9353	0.9369
CASTER [47]		0.8676	0.9268	0.829	0.8767
PTB-DDI [37]		0.86	0.896	0.8221	0.825
Mol2Image	ChCh-Miner	**0.9683**	**0.9989**	0.9747	**0.9699**
1D CNN [54]		0.9492	0.9943	0.9529	0.9495
CASTER [47]		0.9073	0.9956	**0.9993**	0.9448
PTB-DDI [37]		0.935	0.983	0.945	0.932

Bold values indicate the best predictive performance (the highest results) achieved among all the compared models for each respective evaluation metric

Overall, these comparative results highlight the consistent superiority of the proposed model Mol2Image across different datasets and evaluation metrics. The performance gains can be attributed to the model’s ability to learn rich, deep structural representations through the image-based embedding of molecular data. Additionally, the integration of contextual chemical information may have further enhanced the model’s capacity to generalize across drug types and interaction patterns.

## Discussion

The experimental results demonstrate that Mol2Image consistently outperforms existing deep learning models. This superiority is largely attributed to the model’s exclusive reliance on intrinsic chemical structures rather than external clinical data. Consequently, Mol2Image provides a highly generalizable framework capable of screening interactions based strictly on molecular descriptors, circumventing the need for extensive clinical records. Specifically, Mol2Image achieved better performance than PTB-DDI [37] and CASTER [47]. While both baselines rely solely on 1D SMILES strings, Mol2Image extracts deeper physicochemical properties through its optimized molecular descriptors before transforming them into structured 2D image representations. This enriched feature mapping enables the model to capture more discriminative interaction patterns. Furthermore, when evaluated on the same benchmarks as StructNet-DDI [42], which utilizes only 13 molecular features, Mol2Image demonstrates significant improvements. Our richer and systematically optimized descriptor space allows the network to learn finer, more detailed structural relationships between drugs. Finally, the proposed model was evaluated against a 1D CNN baseline [54], which generates molecular descriptors but processes them strictly in 1D without image conversion. Because CNN architectures are inherently designed to extract spatial hierarchies and local feature correlations, our 2D image-based representation enables much more effective spatial feature learning than direct 1D processing, ultimately resulting in enhanced predictive accuracy [55]. While the framework demonstrates strong performance independent of clinical data, the current evaluation relies on standard interaction-level splits using approved drugs. A key limitation is the absence of a ‘cold-start’ scenario; therefore, future work will focus on implementing leave-one-drug-out cross-validation to rigorously simulate and evaluate predictions for strictly unseen ‘new drugs’. Additionally, the model can be extended to a multi-class or multi-label prediction task to predict not only the existence of interactions but also the specific interaction types, mechanisms of action, and side effects associated with drug combinations. Incorporating multimodal data such as drug targets, pathways, and clinical records could also enhance the robustness and real-world applicability of the proposed approach. Finally, this feature space can be further enriched by incorporating quantum chemical properties to capture deeper electronic structures. For instance, approaches such as LiTENexus [56] have explored mapping molecules into quantum chemical latent spaces using cross-molecular attention mechanisms. Integrating similar quantum-level representations alongside our 2D spatial mapping could provide complementary insights, potentially enhancing the model’s generalization ability for structurally similar compounds and complex interaction mechanisms.

## Conclusion

This study proposes a novel model for drug–drug interaction (DDI) prediction. The proposed model Mol2Image doesn’t depend on drug clinical data, but it relies on the drug chemical structure. The proposed model Mol2Image outperformed several existing models using benchmark datasets. It incorporates a broader range of molecular descriptors that significantly improve its predictive performance as it uses basic molecular properties, atomic composition & counts, topological descriptors, structural descriptors, physicochemical & electronic Properties, hybridization features, and structural fingerprint descriptors. This made the proposed model study various types of drug descriptors. The proposed approach is evaluated across two benchmark datasets, showing consistent high accuracy. These results highlight the value of combining CNNs with image-based molecular representation. The proposed model, Mol2Image, uses a greater variety of molecular descriptors and a convolutional neural network (CNN) architecture. SMILES have been utilized as representations to extract molecular descriptors, which were then transformed into grayscale images. These images served as input to the CNN, enabling the model to learn complex molecular features and predict potential drug interactions. The proposed model Mol2Image has been compared to other existing models and outperformed these models in accuracy, AUC-ROC, precision, and F1 score, where it achieved the following results on the DrugBank dataset: 0.9608, 0.9907, 0.9612, and 0.9608, respectively, while it achieved the following results on ChCh-Miner: 0.9683, 0.9989, 0.9747, and 0.9699, respectively. In summary, Mol2Image provides a highly efficient, structure-centric solution for DDI prediction that operates entirely independently of clinical data. By integrating an optimized 21-descriptor set with a lightweight, Siamese-like CNN, the framework minimizes computational overhead and spatial noise without sacrificing predictive power. Ultimately, this scalable approach outperforms existing methods and serves as an ideal tool for the preliminary screening of compounds based solely on their molecular structures.

## Data Availability

The ChCh-Miner dataset used in this study was originally obtained from the BioSNAP biomedical network dataset repository (https://snap.stanford.edu/biodata/datasets/10001/10001-ChCh-Miner.html) and was accessed via the MIRACLE [58] benchmark repository at https://github.com/isjakewong/MIRACLE. The DrugBank dataset was originally sourced from the DrugBank database (https://go.drugbank.com/) and was accessed via the PTB-DDI [37] repository at https://github.com/drunkprogrammer/PTB-DDI.
